# Supplementary material for: Splice-Junction-Based Mapping of Alternative Isoforms in the Human Proteome
Source: Cell Rep. Author manuscript; Available in PMC 2020 Jan 15. (PMC6961840; doi:10.1016/j.celrep.2019.11.026)
Supplement: 2 [file NIHMS1546469-supplement-2.zip › DF1/PXD009737/TestisTrypsin/TestisTrypsin_56_LDHA_SADTLWGIQKELQG.pdf]

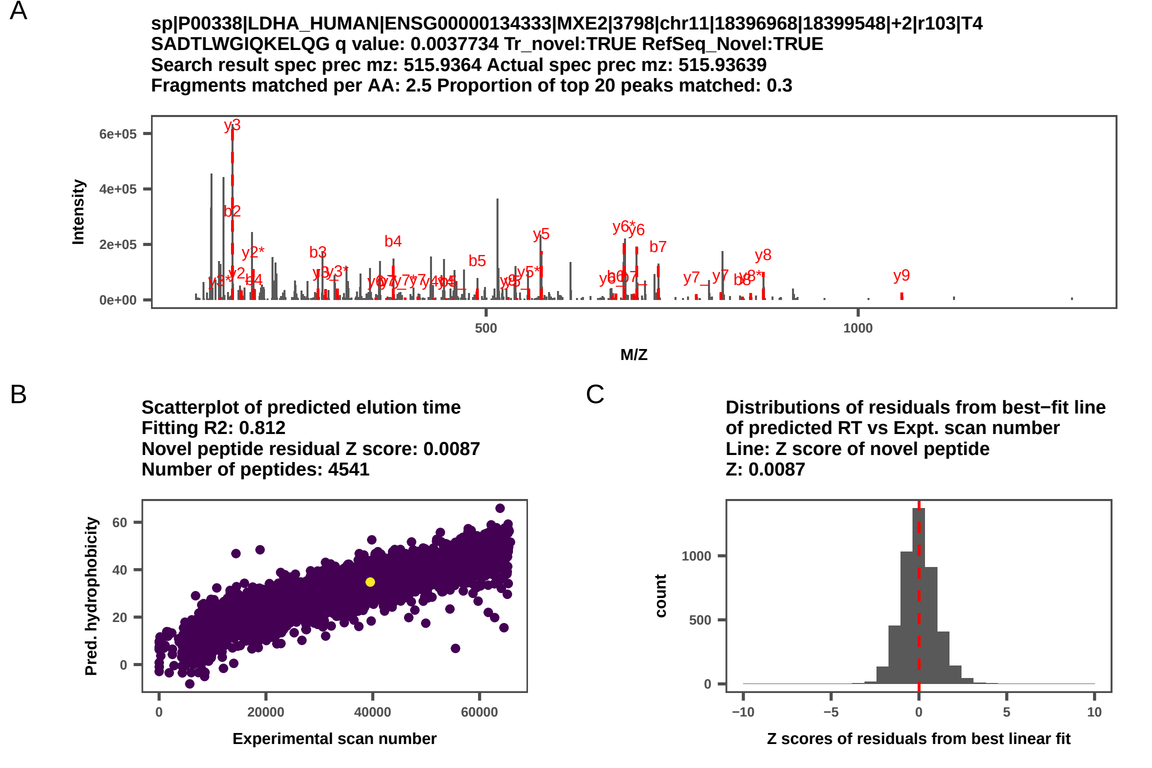
A
sp|P00338|LDHA_HUMAN|ENSG00000134333|MXE2|3798|chr11|18396968|18399548|+2|r103|T4
SADTLWGIQKELQG q value: 0.0037734 Tr_novel:TRUE RefSeq_Novel:TRUE
Search result spec prec mz: 515.9364 Actual spec prec mz: 515.93639
Fragments matched per AA: 2.5 Proportion of top 20 peaks matched: 0.3
Intensity
6e+05
4e+05
2e+05
0e+00
y3
b2
y2*
b3
b4
b5
y5
y6*
y6
b7
y8
y9
500
1000
M/Z
B
Scatterplot of predicted elution time
Fitting R2: 0.812
Novel peptide residual Z score: 0.0087
Number of peptides: 4541
Pred. hydrophobicity
60
40
20
0
0
20000
40000
60000
Experimental scan number
C
Distributions of residuals from best−fit line
of predicted RT vs Expt. scan number
Line: Z score of novel peptide
Z: 0.0087
count
1000
500
0
−10
−5
0
5
10
Z scores of residuals from best linear fit
